# DO OLDER ADULTS BELIEVE THEY ARE AGING SUCCESSFULLY IN THE CIRCUMPOLAR NORTH?

**DOI:** 10.1093/geroni/igad104.3286

**Published:** 2023-12-21

**Authors:** Britteny Howell, Amber Worthington, Dale Golden, Leslie Redmond

**Affiliations:** University of Alaska Anchorage, Anchorage, Alaska, United States; University of Alaska Anchorage, Anchorage, Alaska, United States; University of Alaska Anchorage, Anchorage, Alaska, United States; University of Manitoba, Winnipeg, Manitoba, Canada

## Abstract

People’s perspectives on successful aging may impact their own aging outcomes. To gain more insight, we surveyed a convenience sample of 58 adults aged 55+ years in urban Alaska about their perceptions of their own aging using Fowler’s successful aging scale. Overall, older adults rated their aging in the middle of the 1-7 scale across all three questions: how well they are aging, how “successfully” they have aged up to now, and how they rate their lives these days (M=4.17, SD=1.16, range=1-7 where 1 = not at all successful and 7 = extremely successful). Correlational analyses indicated that as perceptions of successful aging increase, self-reported good general health (M=2.90, SD=1.00, range=1=5) increases, r(56)=.499, p<.001, and self-reported disability (M=0.71, SD=0.92, range=0-3) decreases, r(56)=-.27, p<.05. Despite a small sample size, these findings that perceptions of successful aging increased as general heath increased and self-reported disability decreased, suggest that participants may perceive successful aging and good general health to be positively correlated. Perceptions of successful aging were not related to age in this sample (M=71.98, SD=8.74), r(56)=.125, p=.349. This is promising, as it may indicate that older adults recognize good health and absence of disability as integral components for successful aging regardless of chronological age providing opportunities for targeted messaging in future interventions. Future work will explore opportunities to promote successful aging and overcome some of the challenges related to healthy aging in this urban location, including the Circumpolar environment, historical trauma of Indigenous peoples, and a lack of accessible healthcare across Alaska.

